# COVID-19 Epidemic: Possibility of Artificial Intelligence in Infection Control and Prevention

**DOI:** 10.2188/jea.JE20200203

**Published:** 2020-08-05

**Authors:** Zhao-Yao Qi, Pei-Yuan Zhao, Shao-Hui Geng, Huai-Min Yi, Li-Ping Yang

**Affiliations:** 1 Basic Medical College, Henan University of Chinese Medicine, Zhengzhou, China; 2 First Clinical Medical College, Henan University of Chinese Medicine, Zhengzhou, China

**Keywords:** control and prevention, infectious diseases, artificial intelligence, public health

Over the past 17 years, new infectious diseases have become more frequent and serious, posing a global threat. Severe Acute Respiratory Syndrome swept through more than 30 countries in 2002, infecting 8,098 people and killing 774.^1^ It is estimated that influenza causes about 3 to 5 million cases worldwide every year.^2^ Ebola virus disease has appeared in more than a dozen countries since 1976, with a total of 31,161 infections and 12,999 deaths.^3^ As of February 2020, novel coronavirus (COVID-19) has affected 27 countries, with a reported 75,748 confirmed cases and 2,121 deaths.^4^ The World Health Organization has declared it an emergency public health event.

The traditional control and prevention system has encountered unprecedented challenges. For example, in Wuhan, traditional methods of prevention and control were mainly used. The number of confirmed cases and deaths from COVID-19 has increased 20 times in just over 20 days, reaching 45,027 cases,^5^ and the situation is still grim. However, Shanghai, an international metropolis with a population of 24 million, relies on an artificial intelligence (AI) analysis platform provided by Wei Zhi Technology using location-based service^5^ to monitor and analyze the floating population and formulate scientific prevention and control strategies. As of February 19, only 333 cases have been confirmed in Shanghai, with 2 deaths.^6^

It may be possible to use AI to build a new infectious disease control and prevention system that would work across the whole country or even the globe. It could be key to dealing with current and future outbreaks. At the national level, AI, mega data platforms, and epidemiological investigations are unified. Dynamic model analysis of virus transmission is conducted based on spatial mega-data, and location mega-data are used to predict when people will come into contact with high-risk transmission areas. These systems can be used for regional retrospective epidemic source (confirmation of suspected sources), early warning for both high-risk areas and close contact, and both monitoring and handling of the epidemic. AI may be used to provide decision support for disease prevention and control, to provide early warning for ordinary people, and to predict the course of the epidemic according to global traffic data. This could be of great significance for global epidemic prevention and control. A disease prevention and control system like these could affect the course of human history.

## References

[1] Chang D, Xu H, Rebaza A, Protecting health-care workers from subclinical coronavirus infection. Lancet Respir Med. 2020;8(3):e13. 10.1016/S2213-2600(20)30066-7 32061333 PMC7128440

[2] WHO. Fact sheets, Influenza (Seasonal). 6 November 2018. Available at: http://www.who.int/news-room/fact-sheets/detail/influenza-(seasonal) (accessed May 18, 2020).

[3] WHO. Ebola virus disease. 10 February 2020. Available at: http://www.who.int/news-room/fact-sheets/detail/ebola-virus-disease (accessed May 18, 2020).

[4] WHO. Novel coronavirus (2019-nCoV) situation report - 31. Feb 20, 2020. Available at: http://www.who.int/docs/default-source/coronaviruse/situation-reports/20200220-sitrep-31-covid-19.pdf?sfvrsn=dfd11d24_2 (accessed May 18, 2020).

[5] Wei Zhi Technology (WAYZ). Available at: http://www.wayz.ai/newscenterdetails/1 (accessed May 18, 2020).

[6] China Shanghai Municipal Health Commission. Announcement of national health commission of Shanghai. February 19, 2020. Available at: http://wsjkw.sh.gov.cn/xwfb/20200219/e42cbeae29d94b5eabf3113d69a2bba7.html (accessed May 18, 2020).

